# Induction chemoimmunotherapy may achieve non-inferior outcomes to consolidation immunotherapy in patients with unresectable stage III NSCLC: a real-world multicenter retrospective study

**DOI:** 10.3389/fimmu.2025.1591134

**Published:** 2025-06-12

**Authors:** Song Guan, Yu Wang, Qing Liu, Meng Yan, Kai Ren, Jun Wang, Nan Bi, Lujun Zhao

**Affiliations:** ^1^ Department of Radiation Oncology, Tianjin Medical University Cancer Institute & Hospital, National Clinical Research Center for Cancer, Tianjin’s Clinical Research Center for Cancer, Key Laboratory of Cancer Immunology and Biotherapy, Tianjin, China; ^2^ Department of Radiation Oncology, Beijing Tuberculosis and Thoracic Tumor Research Institute/Beijing Chest Hospital, Cancer Research Center, Capital Medical University, Beijing, China; ^3^ Department of Radiation Oncology, National Cancer Center/National Clinical Research Center for Cancer/Cancer Hospital, Chinese Academy of Medical Sciences and Peking Union Medical College, Beijing, China; ^4^ Department of Radiotherapy, The Fourth Hospital of Hebei Medical University, Hebei Clinical Research Center for Radiation Oncology, Shijiazhuang, China

**Keywords:** chemoradiotherapy, consolidation immunotherapy, induction chemoimmunotherapy, non-small cell lung cancer, prognosis

## Abstract

**Background:**

Consolidation immunotherapy after chemoradiotherapy (CRT) is the standard of care for unresectable stage III non-small cell lung cancer (NSCLC). However, the role of upfront chemoimmunotherapy before CRT remains unclear. This study aims to investigate the value of induction chemoimmunotherapy before CRT in unresectable stage III NSCLC.

**Methods:**

Unresectable stage III NSCLC patients who received induction chemoimmunotherapy before CRT or consolidation immunotherapy after CRT from four centers were retrospectively enrolled. The Kaplan-Meier method was used to estimate progression-free survival (PFS) and overall survival (OS), and one-to-one propensity score matching (PSM) was used to further minimize confounding.

**Results:**

A total of 262 patients were enrolled, with 124 (47.3%) receiving induction chemoimmunotherapy (Ind group) and 138 (52.7%) receiving consolidation immunotherapy (Con group). Further 1:1 PSM analysis showed that induction chemoimmunotherapy achieved comparable outcomes to consolidation immunotherapy (2-year PFS: 56.0% vs. 45.6%, P=0.327; 2-year OS: 81.0% vs. 79.2%, P=0.960) with fewer cycles of immunotherapy (median 4 vs. 10 cycles, P<0.001). The incidence of treatment-related adverse events was similar (P>0.05). Exploratory analysis found that patients with < 4 cycles of induction immunotherapy had similar PFS (median NR vs. 30.1 months, 2-year PFS 50.8% vs. 54.4%, P=0.932) but prolonged OS (median NR vs. 46.0 months, 2-year OS 89.0% vs. 75.5%, P=0.112) compared to those with ≥ 4 cycles of induction immunotherapy.

**Conclusion:**

Upfront chemoimmunotherapy before CRT appears to be feasible and safe, and may achieve non-inferior outcomes to consolidation immunotherapy with fewer cycles of immunotherapy.

## 1 Introduction

The treatment paradigm for stage III non-small cell lung cancer (NSCLC) has been revolutionized by the advent of immunotherapy. For patients with unresectable stage III NSCLC, the current standard of care is consolidation immunotherapy following concurrent chemoradiotherapy (cCRT), known as the PACIFIC regimen (1, 2), although the optimal combination strategy of immunotherapy and chemoradiotherapy (CRT) remains unclear. The 5-year progression-free survival (PFS) rate in the PACIFIC trial was only 33.1%, indicating that nearly 70% of patients with stage III NSCLC had uncontrolled disease (2). Besides, not only is the proportion of patients receiving consolidation immunotherapy low, but the proportion of patients receiving cCRT is also low for various reasons, such as excessive target volume or poor tolerability (3, 4). Although the addition of consolidation immunotherapy after sequential CRT (sCRT) may benefit patients with stage III NSCLC, it is not as effective as consolidation immunotherapy after cCRT (5, 6). Therefore, optimizing the combination of CRT and immunotherapy is urgently needed. In the context of surgery, immunotherapy, whether used preoperatively or postoperatively, confers a survival benefit in patients with resectable NSCLC (7–9), raising the question of whether upfront chemoimmunotherapy prior to CRT could benefit patients or achieve non-inferior outcomes to consolidation immunotherapy in patients with unresectable stage III NSCLC. However, apart from a few single-arm clinical trials or retrospective studies that have demonstrated the safety and efficacy of upfront immunotherapy before CRT based on the use of consolidation immunotherapy, there are currently no studies to answer this question (10–12). Therefore, we conducted this multicenter retrospective study to investigate the role of upfront chemoimmunotherapy before CRT in unresectable stage III NSCLC through real-world data.

## 2 Materials and methods

### 2.1 Patient selection

Patients with unresectable stage III NSCLC who received induction chemoimmunotherapy before CRT without consolidation immunotherapy, or consolidation immunotherapy after CRT at Tianjin Medical University Cancer Institute & Hospital, National Cancer Center/National Clinical Research Center for Cancer/Cancer Hospital, Beijing Chest Hospital, and the Fourth Hospital of Hebei Medical University between February 2018 and May 2023 were enrolled. The inclusion criteria for this study consisted of the following: 1) patients aged 18 years or older; and 2) individuals with histopathologically confirmed stage III NSCLC, and 3) received induction chemoimmunotherapy before thoracic radiation or consolidation immunotherapy after thoracic radiation. The exclusion criteria encompassed: 1) patients exhibiting an anaplastic lymphoma kinase (ALK) rearrangement or an epidermal growth factor receptor (EGFR) mutation; 2) those with a history of any prior cancer-specific treatment; 3) patients who experienced tumor progression before immunotherapy; 4) individuals who received induction immune checkpoint inhibitor (ICI) monotherapy; and 5) patients who received immunotherapy before and after radiotherapy, or concurrently with radiotherapy, or as part of a clinical trial. The medical records were utilized to extract baseline characteristics and therapeutic information pertinent to the patients. The World Health Organization (WHO) criteria (13) and the 8th edition classification by the International Association for the Study of Lung Cancer (14) were employed to determine the histological type and stage of each NSCLC case.

According to the sequencing of immunotherapy and radiotherapy, patients were divided into the induction chemoimmunotherapy (Ind), and consolidation immunotherapy (Con) groups, respectively. This study conformed to the provisions of the Declaration of Helsinki (as revised in 2013) and was approved by the institutional medical ethics committee (No. bc2022212).

### 2.2 Drug treatment

The treatment scheme for each patient was decided by a multidisciplinary team including radiation oncologists and surgeons. Induction chemoimmunotherapy is given mainly to reduce the target volume that is too large or too extensive to meet the normal tissue constraints for definitive CRT, or to downstage as far as possible, thereby increasing the possibility of radical resection. The PD-1/PD-L1 inhibitors used included Atezolizumab, Camrelizumab, Durvalumab, Nivolumab, Pembrolizumab, Sintilimab, Sugemalimab, Tislelizumab and Toripalimab. These nine kinds of ICIs have been approved for the treatment of locally advanced or metastatic NSCLC based on the promising outcomes in NSCLC patients (1, 5, 7, 11, 15–19). Different chemotherapy regimens were administrated to each patient based on various factors, including the histological type of the tumor and the individual clinical condition of the patient.

### 2.3 Study outcomes

Clinical outcomes were evaluated, including progression-free survival (PFS) and overall survival (OS). PFS was calculated from the initiation of treatment until the first recorded instance of disease progression, the date of death without progression, or the last follow-up. OS was estimated from the start of treatment until death or the last follow-up. Patients underwent follow-up visits every three months for the first two years and thereafter every six months, encompassing clinical evaluations, computed tomography (CT) or positron emission tomography (PET) scans, along with additional investigations when clinically warranted. Treatment-related adverse events (TRAEs) for individual patients were evaluated using the Common Terminology Criteria for Adverse Events (CTCAE) version 5.0. Objective response rates (ORRs) and disease control rates (DCRs) were assessed following induction immunotherapy, where ORR was defined as the sum of partial response (PR) and complete response (CR), and DCR was defined as the sum of PR, CR, and stable disease (SD) in accordance with the Response Evaluation Criteria in Solid Tumors (RECIST) v1.1.

### 2.4 Statistical analysis

Demographic and clinical characteristics of patients between treatment arms were compared using the Wilcoxon ranked sum test for continuous variables and the Chi-square test or Fisher’s exact test for categorical variables. Survival analyses were performed using the Kaplan-Meier method to estimate PFS and OS, which were then compared using the log-rank test. When the univariate analysis yielded a P value of ≤ 0.1, the variable was incorporated into the multivariate Cox regression analysis. Subgroup analysis (age [< 65 years or ≥ 65 years], sex [male or female], WHO histology type [squamous, non-squamous, or not otherwise specified], cancer stage [IIIA, IIIB, or IIIC], chemoradiotherapy modality [sequential or concurrent], radiotherapy dose [< 54 Gy or ≥ 54 Gy], smoking history [never, former or current], ECOG performance status [0, 1 or 2]) for PFS was performed to assess the consistency of treatment effects in patient subgroups. Subgroup analysis used an unstratified Cox proportional hazards model with treatment as a covariate. To minimize confounding, one-to-one propensity score matching (PSM) was performed with a caliper setting of 0.02, adjusting for various baseline characteristics. Statistical significance was set at a P value of less than 0.05. All statistical calculations were performed using SPSS software, version 25 (IBM Corporation, Armonk, NY, USA).

## 3 Results

### 3.1 Baseline characteristics

**Figure 1:**
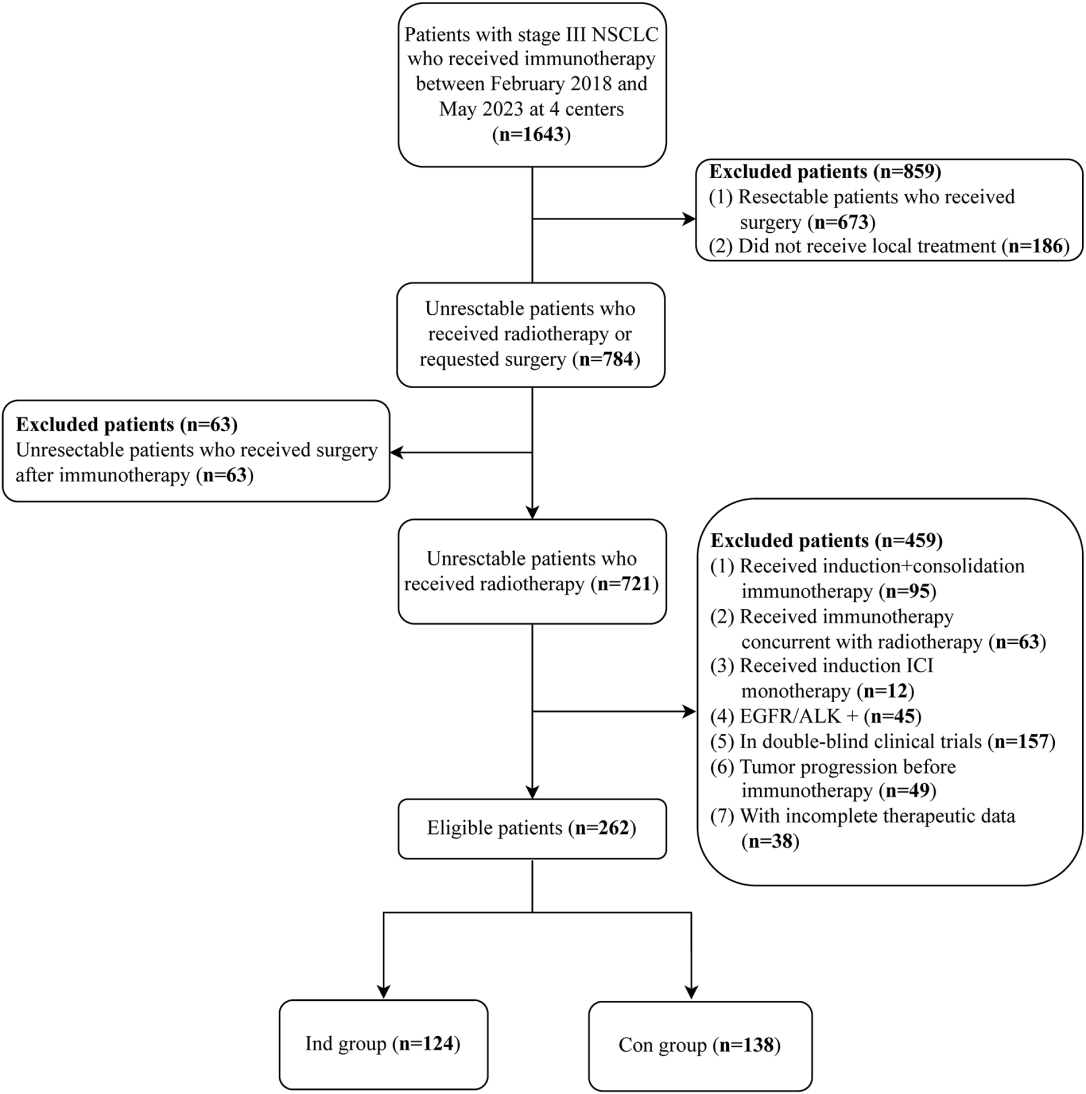
Patient inclusion flow chart.

A total of 262 consecutive eligible patients were enrolled in this study. Among them, 124 (47.3%) received induction chemoimmunotherapy, and 138 (52.7%) received consolidation immunotherapy (**Figure 1**). The median age was 64 years (range 31-79). Patients in the Ind group had more advanced T stage and poorer ECOG performance status compared to those in the Con group. The detailed clinical characteristics of the patients are shown in **Table 1**.

**Table 1:** Baseline demographic and clinical characteristics of patients.

Characteristics	Before PSM		P	After PSM		P
	Ind (n=124)	Con (n=138)		Ind (n=66)	Con (n=66)	
	No. (%)	No. (%)		No. (%)	No. (%)	
Age						
< 65	62 (50.0)	81 (58.7)	0.158	31 (47.0)	36 (54.5)	0.384
≥ 65	62 (50.0)	57 (41.3)		35 (53.0)	30 (45.5)	
Sex						
Male	108 (87.1)	120 (87.0)	0.973	58 (87.9)	59 (89.4)	0.784
Female	16 (12.9)	18 (13.0)		8 (12.1)	7 (10.6)	
WHO histology						
Squamous	93 (75.0)	85 (61.6)	<0.001	50 (75.8)	50 (75.8)	1.000
Non-squamous	26 (21.0)	53 (38.4)		16 (24.2)	16 (24.2)	
NOS	5 (4.0)	0 (0.0)		0 (0.0)	0 (0.0)	
T stage						
1	6 (4.8)	18 (13.0)	0.015	2 (3.0)	5 (7.6)	0.439
2	30 (24.2)	47 (34.1)		19 (28.8)	24 (36.4)	
3	34 (27.4)	28 (20.3)		15 (22.7)	11 (16.7)	
4	54 (43.5)	45 (32.6)		30 (45.5)	26 (39.4)	
N stage						
0	7 (5.6)	7 (5.1)	0.194	3 (4.5)	5 (7.6)	0.485
1	12 (9.7)	7 (5.1)		5 (7.6)	6 (9.1)	
2	70 (56.5)	70 (50.7)		39 (59.1)	30 (45.5)	
3	35 (28.2)	54 (39.1)		19 (28.8)	25 (37.9)	
Stage						
IIIA	45 (36.3)	48 (34.8)	0.908	26 (39.4)	27 (40.9)	0.732
IIIB	54 (43.5)	64 (46.4)		24 (36.4)	27 (40.9)	
IIIC	25 (20.2)	26 (18.8)		16 (24.2)	12 (18.2)	
CRT modality						
sCRT	92 (74.2)	44 (31.9)	<0.001	39 (59.1)	37 (56.1)	0.725
cCRT	32 (25.8)	94 (68.1)		27 (40.9)	29 (43.9)	
Radiation dose						
< 54 Gy	19 (15.3)	6 (4.3)	0.003	1 (1.5)	3 (4.5)	0.612
≥ 54 Gy	105 (84.7)	132 (95.7)		65 (98.5)	63 (95.5)	
Chemotherapy regimen						
P-based doublet	115 (92.7)	127 (92.0)	0.828	61 (92.4)	62 (93.9)	1.000
Mono-chemo	9 (7.3)	11 (8.0)		5 (7.6)	4 (6.1)	
Smoking						
Never	25 (20.2)	27 (19.6)	0.904	15 (22.7)	11 (16.7)	0.381
Former/Current	99 (79.8)	111 (80.4)		51 (77.3)	55 (83.3)	
ECOG						
0	9 (7.3)	16 (11.6)	0.039	5 (7.6)	3 (4.5)	0.858
1	106 (85.5)	120 (87.0)		60 (90.9)	62 (93.9)	
2	9 (7.3)	2 (1.4)		1 (1.5)	1 (1.5)	

Ind, induction chemoimmunotherapy; Con, consolidation immunotherapy; ECOG, Eastern Cooperative Oncology Group; CRT, chemoradiotherapy; sCRT, sequential chemoradiotherapy; cCRT, concurrent chemoradiotherapy; P-based doublet, platinum-based doublet chemotherapy; Mono-chemo, monochemotherapy; PSM, propensity score matching.

### 3.2 Treatment

The ICI agents used included Atezolizumab (0.7%, n=2), Camrelizumab (11.1%, n=29), Durvalumab (30.9%, n=81), Nivolumab (1.9%, n=5), Pembrolizumab (11.8%, n=31), Sintilimab (24.0%, n=63), Sugemalimab (0.4%, n=1), Tislelizumab (13.7%, n=36), and Toripalimab (5.3%, n=14). Detailed information on the ICI agents in each group is shown in **Supplementary Table S1**. All patients in the Ind group received induction PD-1 inhibitor plus chemotherapy, with a median of 4 cycles of induction immunotherapy (range 1-12). Reasons for patients receiving chemoimmunotherapy before CRT included high tumor burden (70.2%, n=87), ineligibility for cCRT (16.1%, n=20), ineligibility for surgery after preoperative neoadjuvant chemoimmunotherapy (8.9%, n=11), and patient preference (4.8%, n=6). Patients in the Con group received a median of 10 cycles of consolidation immunotherapy (range 1-63). 39.1% (n=54) of patients in the Con group received a PD-1 inhibitor, while 60.9% (n=84) received a PD-L1 inhibitor. More patients in the Con group received cCRT than in the Con group (68.1% vs. 25.8%, P<0.001). For patients receiving sCRT, the cycle numbers of chemotherapy were similar in both groups, with a median of 4 cycles in each (P=0.777).

### 3.3 Efficacy

In the entire cohort, median follow-up from the initiation of treatment was 24.9 months (range 5.1-67.6 months). Median PFS and OS were 25.8 and not reached (NR), respectively. During the investigation, 111 patients developed progressive disease (PD), including 52 (41.9%) and 59 (42.8%) cases in the two groups, respectively. Five (4.0%) patients in the Ind group developed PD during induction chemoimmunotherapy, with 4 patients developing local progression and 1 patient developing systemic progression. Sixty-three patients died at the time of analysis, including 29 (23.4%) and 34 (24.6%) cases in the two groups, respectively. The majority of patients (77.8%) died of lung cancer, and the causes of death for the remaining patients are detailed in **Supplementary Table S2**.

**Figure 2:**
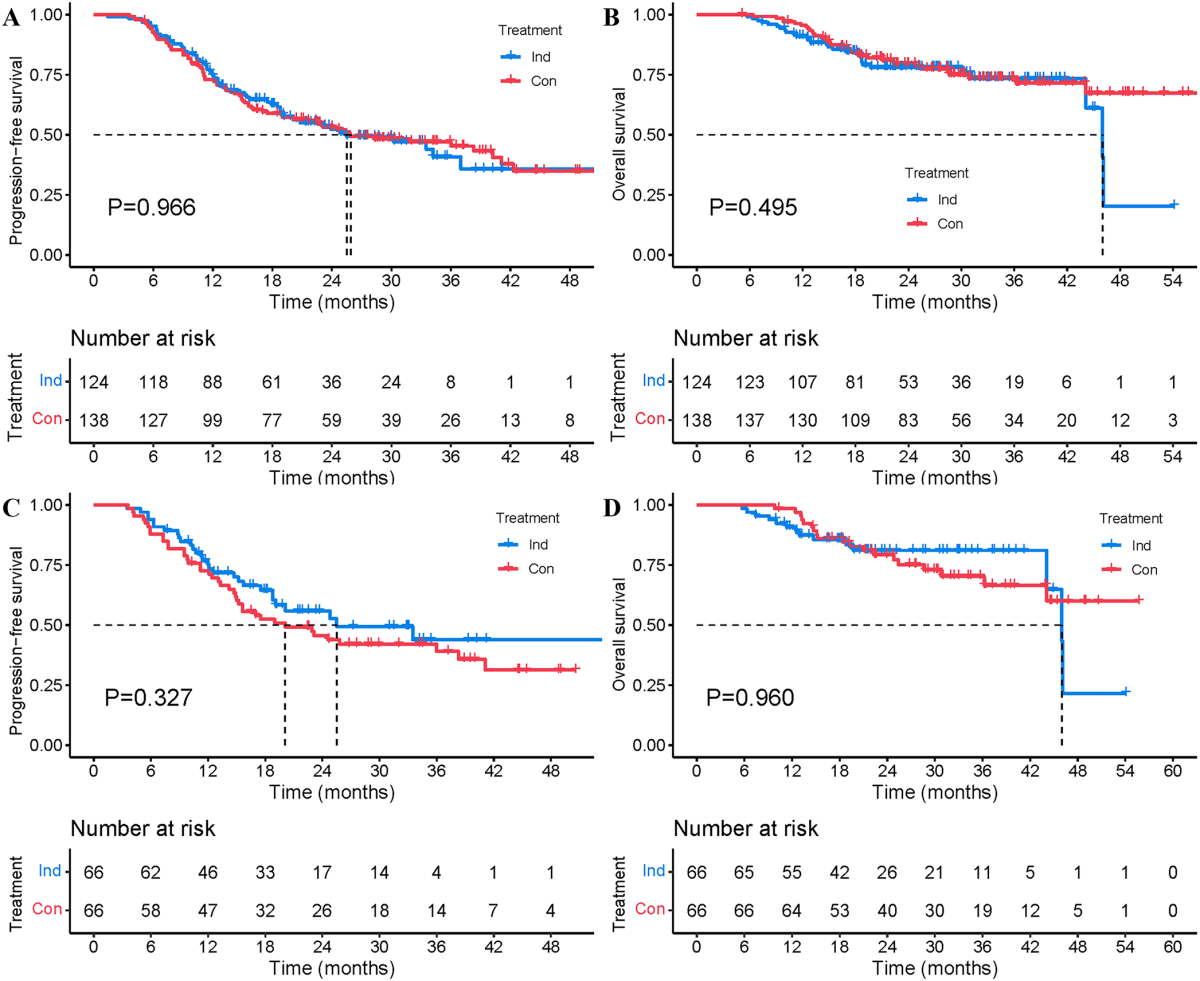
PFS and OS between the Ind and Con groups before and after PSM. **(A)** PFS from the initiation of treatment before PSM. **(B)** OS from the initiation of treatment before PSM. **(C)** PFS from the initiation of treatment after PSM. **(D)** OS from the initiation of treatment after PSM.

**Figure 3:**
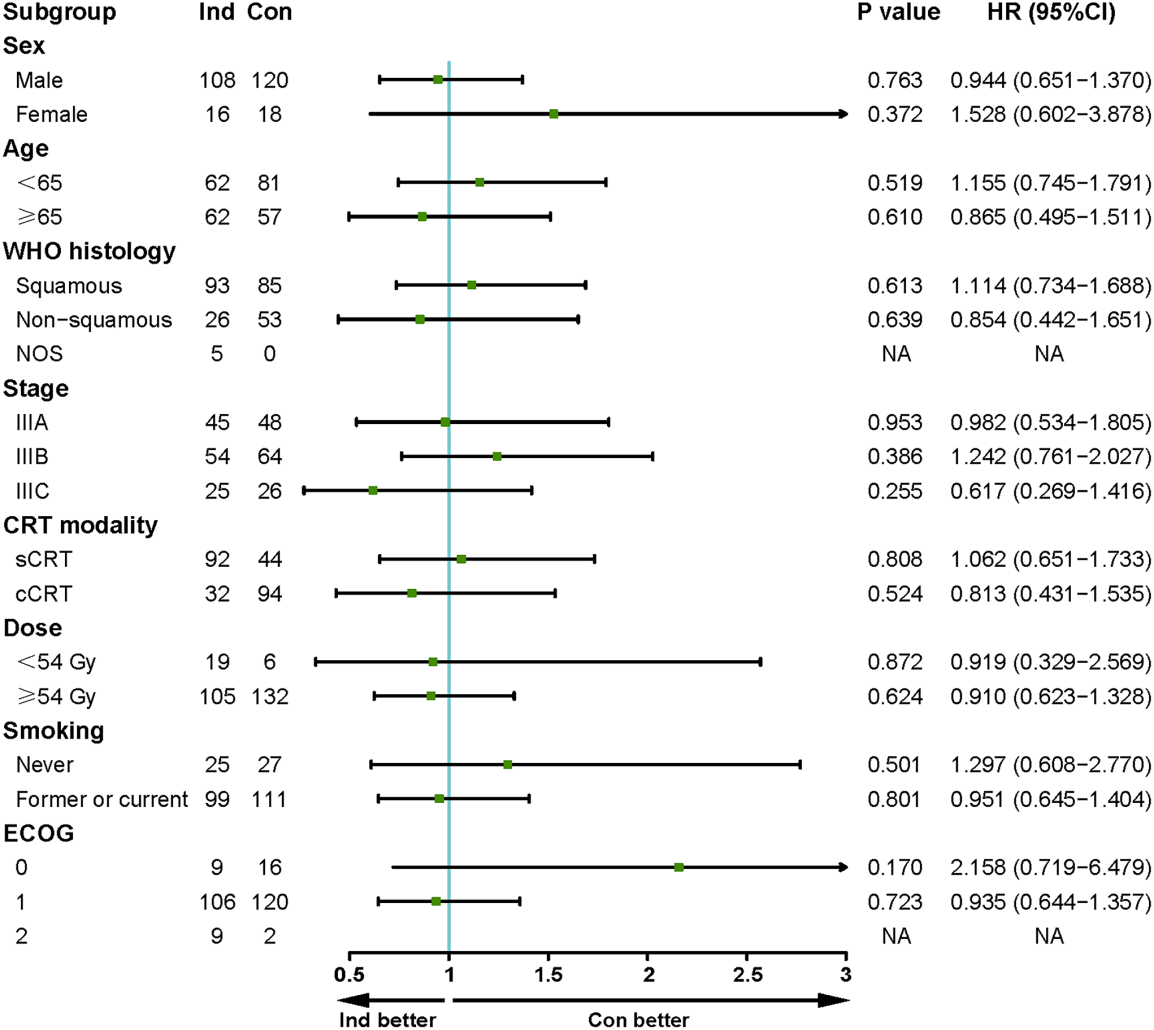
Subgroup analysis of prognostic factors for PFS in the whole population.

The median follow-up for the Ind and the Con groups was 21.5 (range 5.7-54.1) and 28.3 (range 5.1-67.6) months, respectively. Median PFS was 25.5 months in the Ind group vs. 25.9 months in the Con group, with a 1-year PFS rate of 75.5% vs. 72.9% and a 2-year PFS rate of 52.2% vs. 53.1% (P=0.966, **Figure 2A**). Median OS was 46.0 months vs. NR, with a 1-year OS rate of 91.0% vs. 95.6% and a 2-year OS rate of 78.0% vs. 79.5% (P=0.495, **Figure 2B**). Univariate and multivariate analyses further confirmed that sequencing of CRT and immunotherapy was not an independent factor influencing PFS (HR=0.972, P=0.901, **Supplementary Table S3**) and OS (HR=1.400, P=0.340, **Supplementary Table S4**). Similar results were obtained in the multivariate analyses of a subpopulation of patients using PD-1 inhibitors (HR for PFS=0.984, P=0.945; HR for OS=1.509, P=0.257, **Supplementary Tables S5, S6**). Further subgroup analysis demonstrates no advantage in PFS favoring either treatment group across baseline clinicopathological features (**Figure 3**).

After PSM, patients in the Ind group received significantly fewer cycles of immunotherapy than those in the Con group, with a median of 4 cycles vs. 10 cycles (P<0.001). Survival rates were still not significantly different between the two groups. The median PFS was 25.5 months for the Ind group compared to 20.1 months for the Con group, with a 1-year PFS rate of 76.8% vs. 72.7%, and a 2-year PFS rate of 56.0% vs. 45.6% (P=0.327, **Figure 2C**). The median OS was 46.0 months for the Ind group, while it was not reached (NR) for the Con group, with a 1-year OS rate of 90.7% vs. 98.5%, and a 2-year OS rate of 81.0% vs. 79.2% (P=0.960, **Figure 2D**). Patients in the Ind group exhibited a numerically lower incidence of locoregional recurrence (28.8% vs. 40.9%, P=0.144) and distant metastases (18.2% vs. 27.3%, P=0.213) relative to those in the Con group, with no significant differences in the incidence of TRAEs between the two treatment groups (**Table 2**).

**Table 2:** TRAEs between Ind and Con groups.

TRAE	Ind		Con		P
	No.	%	No.	%	
Whole population					
Pneumonitis	68	54.8	92	66.7	0.050
G3/4 pneumonitis	10	8.1	13	9.4	0.699
Esophagitis	32	25.8	58	42.0	0.006
G3/4 esophagitis	1	0.8	2	1.4	1.000
Hematologic toxicity	67	54.0	87	63.0	0.139
G3/4 hematologic toxicity	19	15.3	24	17.4	0.652
Dermatitis	7	5.6	3	2.2	0.254
G3/4 dermatitis	2	1.6	1	0.7	0.926
Matched population					
Pneumonitis	40	60.6	43	65.2	0.589
G3/4 pneumonitis	4	6.1	8	12.1	0.226
Esophagitis	17	25.8	25	37.9	0.135
G3/4 esophagitis	1	1.5	1	1.5	1.000
Hematologic toxicity	35	53.0	40	60.6	0.380
G3/4 hematologic toxicity	12	18.2	9	13.6	0.475
Dermatitis	4	6.1	2	3.0	0.676
G3/4 dermatitis	0	0.0	1	1.5	1.000

Ind, induction chemoimmunotherapy; Con, consolidation immunotherapy; PSM, propensity score matching; TRAE, treatment-related adverse event; G3/4, grade 3/4.

### 3.4 Exploratory analysis

The ORR and DCR after induction chemoimmunotherapy were 66.1% and 96.0%, respectively. Only 4.0% (5/124) patient developed progression during the induction chemoimmunotherapy phase. **Supplementary Figure S1** demonstrates prolonged survival in patients who responded to induction chemoimmunotherapy (CR+PR) compared to those did not (SD+PD) after 1:1 PSM. All clinical characteristics were balanced between groups (**Supplementary Table S7**). Median PFS was 37.0 months in the CR+PR group vs. 15.7 months in the SD+PD group. The 1- and 2-year PFS rates were 76.9% vs. 64.5% and 56.6% vs. 29.3% (P=0.085, **Supplementary Figure S1A**). Median OS were all NR, with a 1-year OS rate of 93.5% vs. 80.6% and a 2-year OS rate of 89.5% vs. 62.1% (P=0.024, **Supplementary Figure S1B**).

**Figure 4:**
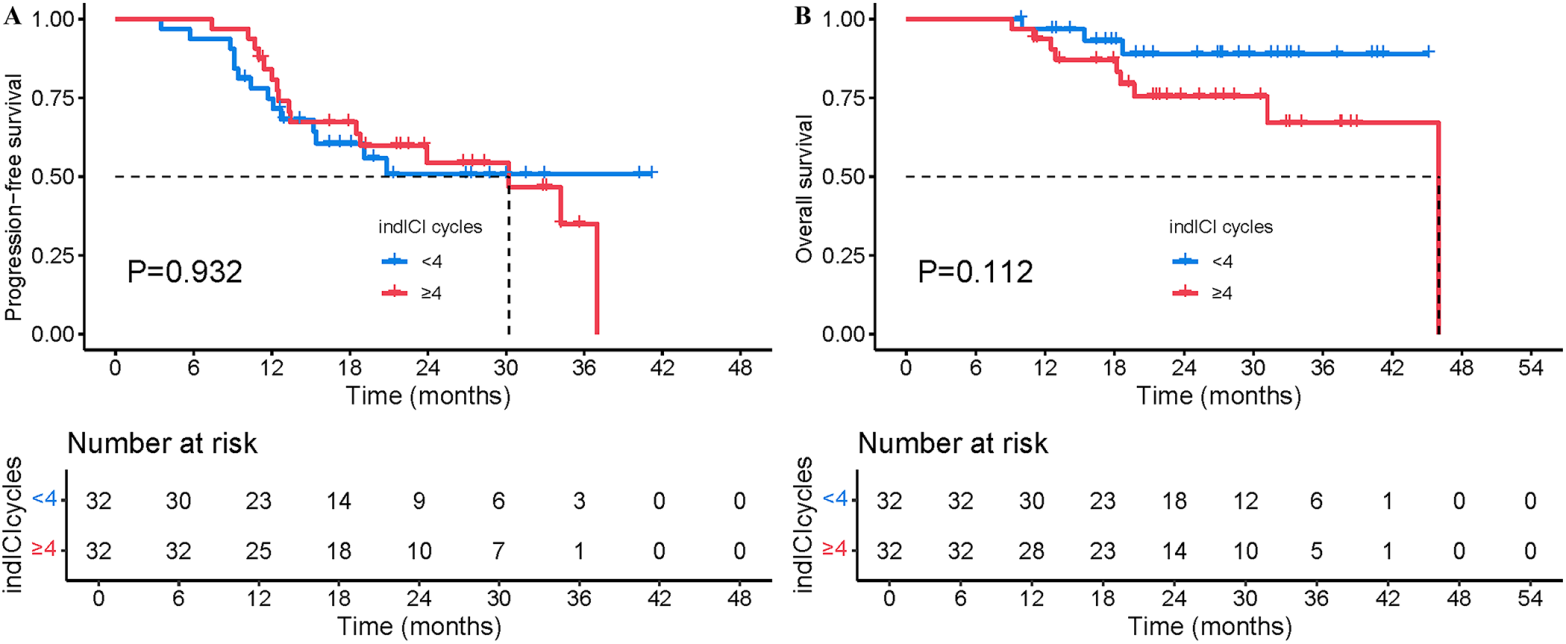
Comparison of PFS and OS between patients who received < 4 cycles of induction immunotherapy and those who received ≥ 4 cycles after PSM. **(A)** PFS from the initiation of treatment after PSM. **(B)** OS from the initiation of treatment after PSM.

The prognostic impact of induction immunotherapy cycles was further investigated. The rationale behind the number of induction immunotherapy cycles chosen was based on patient tolerance (9.7%, n=12), response to treatment (50.8%, n=63), patient refusal (4.0%, n=5) and reference to the preoperative neoadjuvant chemoimmunotherapy modality (35.5%, n=44). According to the median number of induction ICIs cycles, patients were further divided into groups of < 4 and ≥ 4 cycles and matched based on specific criteria including: sex, age, WHO histology, stage, CRT modality, radiation dose, history of smoking, ECOG PS score and response after induction treatment. (**Supplementary Table S8**). After PSM, patients with < 4 cycles of induction immunotherapy exhibited similar PFS (median NR vs. 30.1 months, 2-year PFS 50.8% vs. 54.4%, P=0.932, **Figure 4A**) but prolonged OS (median NR vs. 46.0 months, 2-year OS 89.0% vs. 75.5%, P=0.112, **Figure 4B**) compared to those with ≥ 4 cycles of induction immunotherapy.

## 4 Discussion

Currently, the optimal combination strategy that involves CRT and immunotherapy remains to be fully elucidated. It is unclear whether upfront chemoimmunotherapy prior to CRT could achieve efficacy that is non-inferior to that of consolidation immunotherapy. This study seeks to evaluate the impact of induction chemoimmunotherapy in unresectable stage III NSCLC. To our knowledge, this is the first multicenter study that assesses the role of upfront chemoimmunotherapy before CRT in unresectable stage III NSCLC using real-world data and directly compare it with consolidation immunotherapy.

Although the PACIFIC regimen remains the standard of care for unresectable stage III NSCLC, the significant proportion of patients in the real world who are ineligible for cCRT due to high tumor burden or high risk of pulmonary toxicity, together with the low proportion of patients receiving subsequent consolidation immunotherapy, leading to an increasing recommendation of upfront chemoimmunotherapy before CRT (20, 21). Induction therapy has the theoretical advantages of reducing the target volume to adhere to normal tissue constraints, promoting early intervention for distant micrometastatic disease, and helping to identify the treatment-sensitive population compared to consolidation therapy. Notably, whereas the value of preoperative chemoimmunotherapy in resectable or even potentially resectable NSCLC has been gradually recognized, the role of upfront chemoimmunotherapy before thoracic radiation in unresectable stage III NSCLC has not been extensively explored. At our four centers, induction chemoimmunotherapy is attempted in a proportion of patients with high tumor burden or a strong desire for surgery to shrink the tumor as much as possible. Although all patients who received induction chemoimmunotherapy were advised to continue with consolidation immunotherapy, a significant number of patients declined due to financial burden, concerns about TRAEs, etc., giving us this unique opportunity to explore the value of induction chemoimmunotherapy. The preliminary study found that adding only a median of 4 cycles of immunotherapy before CRT significantly improved PFS in patients with unresectable stage III NSCLC compared with conventional CRT modalities, but this study did not directly compare this treatment regimen with consolidation immunotherapy (22). Interestingly, further PSM analysis in our study indicated that induction chemoimmunotherapy alone may yield outcomes comparable to those achieved with consolidation immunotherapy, with fewer cycles of immunotherapy. The underlying mechanism may be due to the addition of chemotherapy during immunotherapy. Previous studies have shown superior outcomes with chemoimmunotherapy compared to immunotherapy alone, both in advanced NSCLC and in the neoadjuvant phase of early-stage NSCLC (23, 24). In the present study, most patients in the Con group received ICI monotherapy after CRT, while those who received induction immunotherapy all received the combination of immunotherapy and chemotherapy. In addition, a preclinical study suggested that induction immunotherapy is superior to consolidation immunotherapy in reducing distant metastases (25), which may also contribute to this result. Of note, the role of induction chemoimmunotherapy in our study may be underestimated because we excluded patients with disease progression prior to immunotherapy based on the inclusion criteria of the PACIFIC regimen, whereas a significant proportion of patients experience disease progression before consolidation immunotherapy in real-world clinical practice. However, even in this setting, induction chemoimmunotherapy still achieved non-inferior outcomes to consolidation immunotherapy, suggesting the promising outcomes of this new approach.

In addition to efficacy, the safety and feasibility of induction chemoimmunotherapy is also a concern of our study. Only 4.0% of patients who received induction chemoimmunotherapy progressed during the induction immunotherapy phase, and the ORR and DCR after induction immunotherapy were 66.1% and 96.0%, respectively, which is similar to the findings of Wang et al. (76.1% and 86.7%) (26). We further explored the relationship between treatment response and prognosis. Despite various tumor responses to treatment after immunotherapy, such as pseudoprogression and hyperprogression (27, 28), our results demonstrated that tumor shrinkage on imaging after immunotherapy remains an important indicator of improved prognosis. In addition, albeit non-significant, the numerically lower incidence of TRAEs in the Ind group, especially the incidence of pneumonia, which is of greater concern, suggests the safety and feasibility of upfront chemoimmunotherapy before CRT.

The potential theoretical advantages of induction treatment and the remarkable efficacy of preoperative neoadjuvant chemoimmunotherapy have led to the gradual application of induction chemoimmunotherapy in clinical practice. However, the evidence on how many cycles of immunotherapy before CRT are more appropriate is limited. As an exploratory treatment modality, the rationale behind the number of induction immunotherapy cycles chosen is mainly based on the sequential chemoradiotherapy modality to minimize tumor burden, or in reference to preoperative neoadjuvant chemoimmunotherapy cycles. However, even in the surgical setting, the optimal number of cycles of neoadjuvant immunotherapy is controversial. Although 3 cycles of neoadjuvant chemoimmunotherapy appears to be more acceptable, a real-world study found that there was no significant difference in the MPR rate or survival outcomes between 2 cycles and 3–4 cycles of neoadjuvant immunotherapy (29, 30). Conversely, a phase 2 clinical trial demonstrated that 3 cycles of neoadjuvant chemoimmunotherapy archived numerically higher MPR rate (41.4% vs. 26.9%, P=0.260) and pCR rate (24.1% vs. 19.2%, P=0.660) compared with 2 cycles (31). Our result showed that patients with < 4 cycles of induction immunotherapy appeared to have prolonged survival, which is consistent with the findings of Wang et al. (26). They found no difference in ORR between 2 and 4 cycles of induction immunotherapy (60.5% vs. 69.8%, P=0.248), but DCR after 2 cycles was significantly higher than after 4 cycles (97.7% vs. 88.4%, P=0.046). Taken together, early local radical treatment for stage III NSCLC is crucial. Prolonging cycles of induction immunotherapy excessively may not only fail to provide further survival benefits, but may also lead to disease progression and an increased incidence of TRAEs.

The present study has several inherent limitations. Firstly, the retrospective nature of the study limits the extent to which the findings can be generalized. In addition, the study is limited by incomplete data on PD-L1 expression, as this biomarker is not routinely assessed in stage III NSCLC across the four participating centers. Furthermore, the study did not differentiate between various ICIs. Although previous research suggests that there are no significant differences in safety and efficacy profiles among different ICIs or ICI types (32–34), and our study found no significant effect of ICI type on survival outcomes, future investigations should use a uniform ICI and stratify patients based on PD-L1 expression levels to minimize confounding. Finally, the study’s conclusions may be somewhat limited by the relatively modest patient sample size. Despite these limitations, this is the first multicenter study to evaluate the role of upfront chemoimmunotherapy before CRT in unresectable stage III NSCLC using real-world data, and we believe this study may shed some light on clinical practice or provide a treatment option for patients.

## 5 Conclusions

Upfront chemoimmunotherapy before CRT appears to be feasible and safe, and may achieve comparable outcomes to consolidation immunotherapy with fewer cycles of immunotherapy.

## Data Availability

The raw data supporting the conclusions of this article will be made available by the authors, without undue reservation.
