# Supplementary material for: Induction chemoimmunotherapy may achieve non-inferior outcomes to consolidation immunotherapy in patients with unresectable stage III NSCLC: a real-world multicenter retrospective study
Source: Front Immunol. 2025 Jun 12;16:1591134. doi: 10.3389/fimmu.2025.1591134 (PMC12198117; doi:10.3389/fimmu.2025.1591134)
Supplement: Supplementary file 1 [file DataSheet1.docx]

Table S1 Detailed information on the ICI agents in each group

| ICI agent | Whole population | | Matched population | |
| --- | --- | --- | --- | --- |
|  | Ind (n=124) | Con (n=138) | Ind (n=66) | Con (n=66) |
|  | No. (%) | No. (%) | No. (%) | No. (%) |
| Atezolizumab | 0 (0.0) | 2 (1.4) | 0 (0.0) | 2 (3.0) |
| Camrelizumab | 20 (16.1) | 9 (6.5) | 14 (21.2) | 5 (7.6) |
| Durvalumab | 0 (0.0) | 81 (58.7) | 0 (0.0) | 33 (50.0) |
| Nivolumab | 3 (2.4) | 2 (1.4) | 2 (3.0) | 1 (1.5) |
| Pembrolizumab | 28 (22.6) | 3 (2.2) | 17 (25.8) | 2 (3.0) |
| Sintilimab | 45 (36.3) | 18 (13.0) | 17 (25.8) | 12 (18.2) |
| Sugemalimab | 0 (0.0) | 1 (0.7) | 0 (0.0) | 0 (0.0) |
| Tislelizumab | 26 (21.0) | 10 (7.2) | 14 (21.2) | 6 (9.1) |
| Toripalimab | 2 (1.6) | 12 (8.7) | 2 (3.0) | 5 (7.6) |

Abbreviations: Ind, induction chemoimmunotherapy; Con, consolidation immunotherapy.

Table S2 Causes of death

| Cause of death | Ind (n=29) | Con (n=34) |
| --- | --- | --- |
| Lung cancer | 21 (72.4) | 28 (82.4) |
| COVID-19 | 2 (6.9) | 0 (0.0) |
| Chronic disease | 2 (6.9) | 0 (0.0) |
| Pulmonary infection | 1 (3.4) | 0 (0.0) |
| Esophageal tracheal fistula | 1 (3.4) | 1 (2.9) |
| Cerebral infarction | 1 (3.4) | 0 (0.0) |
| Hemoptysis | 1 (3.4) | 1 (2.9) |
| AEs of subsequent treatment | 0 (0.0) | 2 (5.9) |
| Immune-related myositis | 0 (0.0) | 1 (2.9) |
| Immune-related pneumonitis | 0 (0.0) | 0 (0.0) |
| Cerebral Hemorrhage | 0 (0.0) | 1 (2.9) |
| Accident | 0 (0.0) | 0 (0.0) |

Abbreviations: Ind, induction chemoimmunotherapy; Con, consolidation immunotherapy

Table S3 Univariate and multivariate analyses for PFS

| Factor | Univariate | |  | Multivariate | |  |
| --- | --- | --- | --- | --- | --- | --- |
|  | HR | 95% CI | P | HR | 95% CI | P |
| Age |  |  |  |  |  |  |
| <65 | 1.000 (reference) | | | 1.000 (reference) | | |
| ≥65 | 0.697 | 0.491, 0.988 | 0.043 | 0.711 | 0.500, 1.010 | 0.057 |
| Sex |  |  |  |  |  |  |
| Male | 1.000 (reference) | | |  | | |
| Female | 0.996 | 0.606, 1.637 | 0.988 |  |  |  |
| WHO histology |  |  |  |  |  |  |
| Squamous | 1.000 (reference) | | |  | | |
| Non-squamous | 0.888 | 0.615, 1.284 | 0.529 |  |  |  |
| NOS | 0.000 | 0.000 | 0.959 |  |  |  |
| Stage |  |  |  |  |  |  |
| IIIA | 1.000 (reference) | | |  | | |
| IIIB | 1.241 | 0.845, 1.822 | 0.271 |  |  |  |
| IIIC | 1.042 | 0.632, 1.717 | 0.873 |  |  |  |
| CRT modality |  |  |  |  |  |  |
| sCRT | 1.000 (reference) | | |  | | |
| cCRT | 0.926 | 0.658, 1.302 | 0.657 |  |  |  |
| Dose |  |  |  |  |  |  |
| <54Gy | 1.000 (reference) | | | 1.000 (reference) | | |
| ≥54Gy | 0.513 | 0.316, 0.835 | 0.007 | 0.516 | 0.312, 0.851 | 0.010 |
| Smoking history |  |  |  |  |  |  |
| Never | 1.000 (reference) | | |  |  |  |
| Former/current | 0.959 | 0.628, 1.463 | 0.845 |  |  |  |
| ECOG |  |  |  |  |  |  |
| 0 | 1.000 (reference) | | |  | | |
| 1 | 1.009 | 0.569, 1.791 | 0.976 |  |  |  |
| 2 | 0.560 | 0.183, 1.719 | 0.311 |  |  |  |
| Treatment |  |  |  |  |  |  |
| Con | 1.000 (reference) | | | 1.000 (reference) | | |
| Ind | 1.007 | 0.713, 1.423 | 0.966 | 0.972 | 0.622, 1.519 | 0.901 |
| ICI type |  |  |  |  |  |  |
| PD-1 inhibitor | 1.000 (reference) | | | 1.000 (reference) | | |
| PD-L1 inhibitor | 1.017 | 0.707, 1.464 | 0.926 | 1.034 | 0.651, 1.644 | 0.887 |

Abbreviations: PFS, progression-free survival; HR, hazard ratio; NOS, not otherwise specified; CRT, chemoradiotherapy; sCRT, sequential chemoradiotherapy; cCRT, concurrent chemoradiotherapy; ECOG, Eastern Cooperative Oncology Group; Ind, induction chemoimmunotherapy; Con, consolidation immunotherapy; ICI, immune checkpoint inhibitor.

Table S4 Univariate and multivariate analyses for OS

| Factor | Univariate | |  | Multivariate | |  |
| --- | --- | --- | --- | --- | --- | --- |
|  | HR | 95% CI | P | HR | 95% CI | P |
| Age |  |  |  |  |  |  |
| <65 | 1.000 (reference) | | | 1.000 (reference) | | |
| ≥65 | 0.544 | 0.320, 0.924 | 0.024 | 0.539 | 0.316, 0.920 | 0.023 |
| Sex |  |  |  |  |  |  |
| Male | 1.000 (reference) | | |  | | |
| Female | 1.027 | 0.506, 2.084 | 0.942 |  |  |  |
| WHO histology |  |  |  |  |  |  |
| Squamous | 1.000 (reference) | | |  | | |
| Non-squamous | 0.858 | 0.500, 1.470 | 0.577 |  |  |  |
| NOS | 0.000 | 0.000 | 0.975 |  |  |  |
| Stage |  |  |  |  |  |  |
| IIIA | 1.000 (reference) | | |  | | |
| IIIB | 1.334 | 0.755, 2.356 | 0.320 |  |  |  |
| IIIC | 1.213 | 0.588, 2.501 | 0.601 |  |  |  |
| CRT modality |  |  |  |  |  |  |
| sCRT | 1.000 (reference) | | |  | | |
| cCRT | 1.084 | 0.662, 1.778 | 0.748 |  |  |  |
| Dose |  |  |  |  |  |  |
| <54Gy | 1.000 (reference) | | | 1.000 (reference) | | |
| ≥54Gy | 0.428 | 0.223, 0.821 | 0.011 | 0.443 | 0.227, 0.865 | 0.017 |
| Smoking history |  |  |  |  |  |  |
| Never | 1.000 (reference) | | |  |  |  |
| Former/current | 1.164 | 0.607, 2.235 | 0.647 |  |  |  |
| ECOG |  |  |  |  |  |  |
| 0 | 1.000 (reference) | | |  | | |
| 1 | 2.396 | 0.751, 7.644 | 0.140 |  |  |  |
| 2 | 0.726 | 0.075, 6.987 | 0.782 |  |  |  |
| Treatment |  |  |  |  |  |  |
| Con | 1.000 (reference) | | | 1.000 (reference) | | |
| Ind | 1.190 | 0.722, 1.961 | 0.496 | 1.400 | 0.702, 2.791 | 0.340 |
| ICI type |  |  |  |  |  |  |
| PD-1 inhibitor | 1.000 (reference) | | | 1.000 (reference) | | |
| PD-L1 inhibitor | 1.076 | 0.641, 1.828 | 0.781 | 1.367 | 0.675, 2.768 | 0.386 |

Abbreviations: OS, overall survival; HR, hazard ratio; NOS, not otherwise specified; CRT, chemoradiotherapy; sCRT, sequential chemoradiotherapy; cCRT, concurrent chemoradiotherapy; ECOG, Eastern Cooperative Oncology Group; Ind, induction chemoimmunotherapy; Con, consolidation immunotherapy; ICI, immune checkpoint inhibitor.

Table S5 Univariate and multivariate analyses for PFS in patients receiving PD-1 inhibitor

| Factor | Univariate | |  | Multivariate | |  |
| --- | --- | --- | --- | --- | --- | --- |
|  | HR | 95% CI | P | HR | 95% CI | P |
| Age |  |  |  |  |  |  |
| <65 | 1.000 (reference) | | | 1.000 (reference) | | |
| ≥65 | 0.660 | 0.432, 1.006 | 0.053 | 0.693 | 0.453, 1.062 | 0.092 |
| Sex |  |  |  |  |  |  |
| Male | 1.000 (reference) | | |  | | |
| Female | 1.076 | 0.598, 1.938 | 0.807 |  |  |  |
| WHO histology |  |  |  |  |  |  |
| Squamous | 1.000 (reference) | | |  | | |
| Non-squamous | 0.876 | 0.548, 1.399 | 0.578 |  |  |  |
| NOS | 0.000 | 0.000 | 0.959 |  |  |  |
| Stage |  |  |  |  |  |  |
| IIIA | 1.000 (reference) | | |  | | |
| IIIB | 1.280 | 0.798, 2.052 | 0.305 |  |  |  |
| IIIC | 0.896 | 0.476, 1.684 | 0.732 |  |  |  |
| CRT modality |  |  |  |  |  |  |
| sCRT | 1.000 (reference) | | |  | | |
| cCRT | 1.030 | 0.669, 1.586 | 0.894 |  |  |  |
| Dose |  |  |  |  |  |  |
| <54Gy | 1.000 (reference) | | | 1.000 (reference) | | |
| ≥54Gy | 0.466 | 0.275, 0.792 | 0.005 | 0.489 | 0.284, 0.842 | 0.010 |
| Smoking history |  |  |  |  |  |  |
| Never | 1.000 (reference) | | |  |  |  |
| Former/current | 0.895 | 0.534, 1.501 | 0.675 |  |  |  |
| ECOG |  |  |  |  |  |  |
| 0 | 1.000 (reference) | | |  | | |
| 1 | 0.841 | 0.388, 1.823 | 0.661 |  |  |  |
| 2 | 0.504 | 0.147, 1.723 | 0.275 |  |  |  |
| Treatment |  |  |  |  |  |  |
| Con | 1.000 (reference) | | | 1.000 (reference) | | |
| Ind | 1.045 | 0.672, 1.625 | 0.846 | 0.984 | 0.627, 1.545 | 0.945 |

Abbreviations: PFS, progression-free survival; HR, hazard ratio; NOS, not otherwise specified; CRT, chemoradiotherapy; sCRT, sequential chemoradiotherapy; cCRT, concurrent chemoradiotherapy; ECOG, Eastern Cooperative Oncology Group; Ind, induction chemoimmunotherapy; Con, consolidation immunotherapy.

Table S6 Univariate and multivariate analyses for OS in patients receiving PD-1 inhibitor

| Factor | Univariate | |  | Multivariate | |  |
| --- | --- | --- | --- | --- | --- | --- |
|  | HR | 95% CI | P | HR | 95% CI | P |
| Age |  |  |  |  |  |  |
| <65 | 1.000 (reference) | | | 1.000 (reference) | | |
| ≥65 | 0.417 | 0.213, 0.817 | 0.011 | 0.414 | 0.210, 0.816 | 0.011 |
| Sex |  |  |  |  |  |  |
| Male | 1.000 (reference) | | |  | | |
| Female | 1.704 | 0.812, 3.576 | 0.159 |  |  |  |
| WHO histology |  |  |  |  |  |  |
| Squamous | 1.000 (reference) | | |  | | |
| Non-squamous | 0.979 | 0.497, 1.930 | 0.952 |  |  |  |
| NOS | 0.000 | 0.000 | 0.975 |  |  |  |
| Stage |  |  |  |  |  |  |
| IIIA | 1.000 (reference) | | |  | | |
| IIIB | 1.218 | 0.611, 2.427 | 0.575 |  |  |  |
| IIIC | 0.774 | 0.294, 2.038 | 0.604 |  |  |  |
| CRT modality |  |  |  |  |  |  |
| sCRT | 1.000 (reference) | | |  | | |
| cCRT | 1.231 | 0.657, 2.307 | 0.517 |  |  |  |
| Dose |  |  |  |  |  |  |
| <54Gy | 1.000 (reference) | | | 1.000 (reference) | | |
| ≥54Gy | 0.391 | 0.185, 0.830 | 0.014 | 0.446 | 0.209, 0.954 | 0.037 |
| Smoking history |  |  |  |  |  |  |
| Never | 1.000 (reference) | | |  |  |  |
| Former/current | 0.870 | 0.414, 1.827 | 0.713 |  |  |  |
| ECOG |  |  |  |  |  |  |
| 0 | 1.000 (reference) | | |  | | |
| 1 | 1.734 | 0.416, 7.233 | 0.450 |  |  |  |
| 2 | 0.614 | 0.055, 6.821 | 0.691 |  |  |  |
| Treatment |  |  |  |  |  |  |
| Con | 1.000 (reference) | | | 1.000 (reference) | | |
| Ind | 1.495 | 0.750, 2.980 | 0.253 | 1.509 | 0.741, 3.072 | 0.257 |

Abbreviations: PFS, progression-free survival; HR, hazard ratio; NOS, not otherwise specified; CRT, chemoradiotherapy; sCRT, sequential chemoradiotherapy; cCRT, concurrent chemoradiotherapy; ECOG, Eastern Cooperative Oncology Group; Ind, induction chemoimmunotherapy; Con, consolidation immunotherapy.

Table S7 Baseline characteristics between the CR+PR and SD+PD groups

| Characteristics | Before PSM | |  | After PSM | |  |
| --- | --- | --- | --- | --- | --- | --- |
|  | CR+PR (n=82) | SD+PD (n=42) |  | CR+PR (n=31) | SD+PD (n=31) |  |
|  | No. (%) | No. (%) | P | No. (%) | No. (%) | P |
| Age |  |  |  |  |  |  |
| < 65 | 37(45.1) | 25(59.5) | 0.129 | 17(54.8) | 18(58.1) | 0.798 |
| ≥ 65 | 45(54.9) | 17(40.5) |  | 14(45.2) | 13(41.9) |  |
| Sex |  |  |  |  |  |  |
| Male | 75(91.5) | 33(78.6) | 0.043 | 27(87.1) | 26(83.9) | 1.000 |
| Female | 7(8.5) | 9(21.4) |  | 4(12.9) | 5(16.1) |  |
| WHO histology |  |  |  |  |  |  |
| Squamous | 63(76.8) | 30(71.4) | 0.710 | 26(83.9) | 24(77.4) | 0.749 |
| Non-squamous | 16(19.5) | 10(23.8) |  | 5(16.1) | 6(19.4) |  |
| NOS | 3(3.7) | 2(4.8) |  | 0(0.0) | 1(3.2) |  |
| Stage |  |  |  |  |  |  |
| IIIA | 31(37.8) | 14(33.3) | 0.137 | 14(45.2) | 10(32.3) | 0.455 |
| IIIB | 31(37.8) | 23(54.8) |  | 13(41.9) | 18(58.1) |  |
| IIIC | 20(24.4) | 5(11.9) |  | 4(12.9) | 3(9.7) |  |
| CRT modality |  |  |  |  |  |  |
| sCRT | 60(73.2) | 32(76.2) | 0.716 | 18(58.1) | 23(74.2) | 0.180 |
| cCRT | 22(26.8) | 10(23.8) |  | 13(41.9) | 8(25.8) |  |
| Radiation dose |  |  |  |  |  |  |
| < 54 Gy | 6(7.3) | 13(31.0) | 0.001 | 4(12.9) | 4(12.9) | 1.000 |
| ≥ 54 Gy | 76(92.7) | 29(69.0) |  | 27(87.1) | 27(87.1) |  |
| Smoking |  |  |  |  |  |  |
| Never | 15(18.3) | 10(23.8) | 0.469 | 6(19.4) | 6(19.4) | 1.000 |
| Former/Current | 67(81.7) | 32(76.2) |  | 25(80.6) | 25(80.6) |  |
| ECOG |  |  |  |  |  |  |
| 0 | 6(7.3) | 3(7.1) | 0.920 | 3(9.7) | 1(3.2) | 0.582 |
| 1 | 69(84.1) | 37(88.1) |  | 25(80.6) | 28(90.3) |  |
| 2 | 7(8.5) | 2(4.8) |  | 3(9.7) | 2(6.5) |  |

Abbreviations: CR, complete response; PR, partial response; SD, stable disease; PD, progressive disease; ECOG, Eastern Cooperative Oncology Group; CRT, chemoradiotherapy; sCRT, sequential chemoradiotherapy; cCRT, concurrent chemoradiotherapy; PSM, propensity score matching.

Table S8 Baseline characteristics of groups with different induction immunotherapy cycles

| Characteristics | Before PSM | |  | After PSM | |  |
| --- | --- | --- | --- | --- | --- | --- |
|  | < 4 (n=53) | ≥ 4 (n=71) |  | < 4 (n=32) | ≥ 4 (n=32) |  |
|  | No. (%) | No. (%) | *P* | No. (%) | No. (%) | *P* |
| Age |  |  |  |  |  |  |
| < 65 | 28(52.8) | 34(47.9) | 0.586 | 16(50.0) | 11(34.4) | 0.206 |
| ≥ 65 | 25(47.2) | 37(52.1) |  | 16(50.0) | 21(65.6) |  |
| Sex |  |  |  |  |  |  |
| Male | 49(92.5) | 59(83.1) | 0.124 | 30(93.8) | 30(93.8) | 1.000 |
| Female | 4(7.5) | 12(16.9) |  | 2(6.3) | 2(6.3) |  |
| WHO histology |  |  |  |  |  |  |
| Squamous | 43(81.1) | 50(70.4) | 0.166 | 25(78.1) | 26(81.3) | 0.585 |
| Non-squamous | 7(13.2) | 19(26.8) |  | 5(15.6) | 6(18.8) |  |
| NOS | 3(5.7) | 2(2.8) |  | 2(6.3) | 0(0.0) |  |
| Stage |  |  |  |  |  |  |
| IIIA | 25(47.2) | 20(28.2) | 0.100 | 14(43.8) | 11(34.4) | 0.658 |
| IIIB | 19(35.8) | 35(49.3) |  | 11(34.4) | 14(43.8) |  |
| IIIC | 9(17.0) | 16(22.5) |  | 7(21.9) | 7(21.9) |  |
| CRT modality |  |  |  |  |  |  |
| sCRT | 28(52.8) | 64(90.1) | <0.001 | 24(75.0) | 26(81.3) | 0.545 |
| cCRT | 25(47.2) | 7(9.9) |  | 8(25.0) | 6(18.8) |  |
| Radiation dose |  |  |  |  |  |  |
| < 54 Gy | 7(13.2) | 12(16.9) | 0.572 | 3(9.4) | 4(12.5) | 1.000 |
| ≥ 54 Gy | 46(86.8) | 59(83.1) |  | 29(90.6) | 28(87.5) |  |
| Smoking |  |  |  |  |  |  |
| Never | 9(17.0) | 16(22.5) | 0.446 | 5(15.6) | 8(25.0) | 0.351 |
| Former/Current | 44(83.0) | 55(77.5) |  | 27(84.4) | 24(75.0) |  |
| ECOG |  |  |  |  |  |  |
| 0 | 3(5.7) | 6(8.5) | 0.640 | 2(6.3) | 0(0.0) | 0.399 |
| 1 | 45(84.9) | 61()85.9 |  | 26(81.3) | 29(90.6) |  |
| 2 | 5(9.4) | 4(5.6) |  | 4(12.5) | 3(9.4) |  |
| Response |  |  |  |  |  |  |
| CR+PR | 32(60.4) | 80(70.4) | 0.242 | 24(75.0) | 20(62.5) | 0.281 |
| SD+PD | 21(39.6) | 21(29.6) |  | 8(25.0) | 12(37.5) |  |

Abbreviations: ECOG, Eastern Cooperative Oncology Group; CRT, chemoradiotherapy; sCRT, sequential chemoradiotherapy; cCRT, concurrent chemoradiotherapy; PSM, propensity score matching; Response, response after induction immunotherapy; CR, complete response; PR, partial response; SD, stable disease; PD, progressive disease.


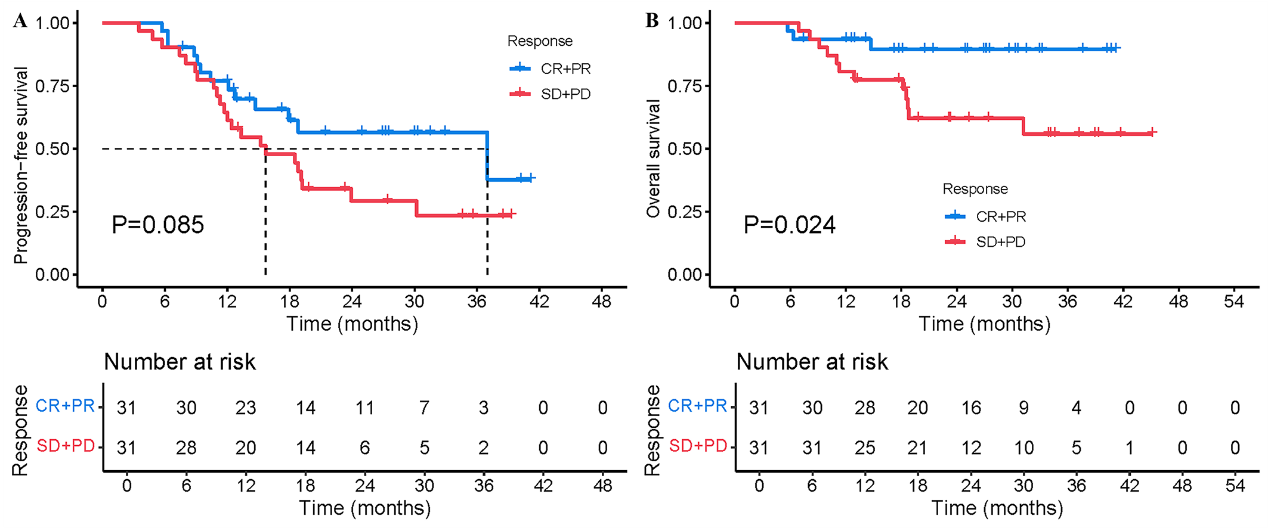


**Figure S1** PFS and OS between the CR+PR and SD+PD groups after PSM. (A) PFS from the initiation of treatment after PSM. (B) OS from the initiation of treatment after PSM.
